# Extracellular vesicle‐associated organotropic metastasis

**DOI:** 10.1111/cpr.12948

**Published:** 2020-11-03

**Authors:** Zhenzhen Mo, Jia Yang Alex Cheong, Lirong Xiang, Minh T. N. Le, Andrew Grimson, Daniel Xin Zhang

**Affiliations:** ^1^ Department of Paediatrics People's Hospital of Guangxi Zhuang Autonomous Region Nanning China; ^2^ Yong Loo Lin School of Medicine National University of Singapore Singapore Singapore; ^3^ Institute for Digital Medicine and Department of Pharmacology Yong Loo Lin School of Medicine National University of Singapore Singapore Singapore; ^4^ Department of Biomedical Sciences Jockey Club College of Veterinary Medicine and Life Sciences City University of Hong Kong Kowloon Hong Kong SAR; ^5^ Department of Molecular Biology and Genetics Cornell University Ithaca NY USA

**Keywords:** cancer, exosomes, extracellular vesicles, metastasis, microvesicles, organotropism

## Abstract

Metastasis refers to the progressive dissemination of primary tumour cells and their colonization of other tissues and is associated with most cancer‐related mortalities. The disproportional and systematic distribution pattern of distant metastasis in different cancers has been well documented, as is termed metastatic organotropism, a process orchestrated by a combination of anatomical, pathophysiological, genetic and biochemical factors. Extracellular vesicles (EVs), nanosized cell‐derived membrane‐bound particles known to mediate intercellular communication, are now considered crucial in organ‐specific metastasis. Here, we review and summarize recent findings regarding EV‐associated organotropic metastasis as well as some of the general mechanisms by which EVs contribute to this important process in cancer and provide a future perspective on this emerging topic. We highlight studies that demonstrate a role of tumour‐derived EVs in organotropic metastasis via pre‐metastatic niche modulation. The bioactive cargo carried by EVs is of diagnostic and prognostic values, and counteracting the functions of such EVs may be a novel therapeutic strategy targeting metastasis. Further investigations are warranted to better understand the functions and mechanisms of EVs in organotropic metastasis and accelerate the relevant clinical translation.

## Graphical abstract

Extracellular vesicles (EVs) mediate organotropic metastasis mainly through pre‐metastatic niche modulation. Primary tumour cells disseminate EVs, which can travel to distant metastatic sites and modulate resident or stroma cells in the pre‐metastatic microenvironment through various pathways and prepare a favourable pre‐metastatic niche.

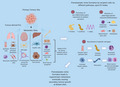

## 1 BACKGROUND

Metastasis is a multistep process in which tumour cells progressively spread from the primary sites of oncogenesis and colonize other tissues or organs.^1^ It is considered one of the most catastrophic hallmarks of cancer.^2^ Most cancer‐related deaths are thought to link to metastasis.^3^ Moreover, clinical observations have documented the disproportional distribution pattern of distant metastasis in various cancers, which is well recognized as metastatic organotropism.^4^ Recently, extracellular vesicles (EVs) and their cargoes are reported to be crucial in organ‐specific metastasis. Here, we concentrate on and summarize the recent findings regarding EV‐associated organotropic metastasis as well as some general mechanisms involved and review the novel translational studies targeting metastasis with EV‐based diagnostic, prognostic and therapeutic strategies before providing a future perspective on this topic.

### 1.1 Multistep mechanism of metastasis

Tumour cells at primary sites of cancer undergo a series of complex but highly regulated processes to spread to and grow in distant organs and tissues. At first, abnormally proliferating tumour cells start to invade surrounding tissues, such as basement membrane, which is frequently accompanied by epithelial to mesenchymal transition and marked by decreased E‐cadherin expression and enhanced N‐cadherin expression.^5^ Next, tumour cells intravasate into blood vessels and begin their journey in the circulation. Inflammatory responses are known to play a crucial role in intravasation by increasing vascular permeability.^6^ Circulating tumour cells in migration are then arrested by adhesive interactions, or neutrophil aggregation or trapped by physical occlusion in vasculature and extravasate.^7^ Upon landing at future metastatic sites, tumour cells resume secondary growth, requiring multiple factors for adaptation to the new environment.^8^ The spatial concept inherent in metastasis, known as the tumour microenvironment, is of rapidly rising interest and relevance to research in cancer biology.^9^

### 1.2 Metastatic routes

Metastasis can happen via various routes, including the lymphatic system, haematogenous routes, transcoelomic spreads, and iatrogenic transplantation. The site‐specific metastases of transcoelomic spreads and iatrogenic transplantation are largely associated with anatomical features. Transcoelomic metastasis occurs when tumour cells disseminate across body cavities such as pericardial, pleural, peritoneal, or subarachnoid spaces.^10^ For instance, the transcoelomic route is the most frequent metastatic pattern in epithelial ovarian malignancy and is responsible for most mortalities.^11^ Similarly, iatrogenic transplantation is caused by carryover of malignant cells during invasive diagnostic or surgical procedures. For this reason, routine biopsy is often not recommended for cases of hepatocellular carcinoma due to the risk of needle‐track seeding.^12^, ^13^ Compared to transcoelomic spreads and iatrogenic transplantation, organ‐specific tropism involved in lymphatic and haematogenous metastasis is more complex and regarded as the results from numerous factors including anatomical and functional characteristics, and molecular and cellular interactions.^14^

### 1.3 Extracellular vesicles

In recent years, EVs have been increasingly recognized as a key player in the tumour microenvironment, with diverse EV cargoes thought to contribute to metastasis and organotropism. Abundant in a variety of human biofluids, from blood^15^ and urine^16^ to cerebrospinal fluid^17^ and semen,^18^ EVs are a class of cell‐derived, membrane‐enclosed nanoparticles secreted by various types of cells,^19^ including cancer cells,^20^ immune cells,^21^ and red blood cells.^22^ Protected by a lipid‐bilayer membrane structure, EVs carry a variety of bio‐functional molecules, both on the membrane and within the vesicles, including proteins, nucleic acids, and lipids.^23^ EV function is largely dependent on the various imparted cargo it shuttles, which can be reflective of the cells of origin.^24^ Based on distinct biogenesis pathways, EVs are typically categorized into three major groups: exosomes, microvesicles and apoptotic bodies.

Exosomes are endosomal in origin and formed by the fusion of multivesicular bodies (MVBs) with plasma membrane. MVBs contain intraluminal vesicles and they are subsequently released into the extracellular lumen when these MVBs fuse with the plasma membrane.^25^ In comparison, microvesicles are generally larger, and they bud directly from the plasma membrane as the molecular cargo is transported to the cell surface. For this reason, they are often referred to as shedding vesicles or shedding bodies. This shedding of microvesicles can be stimulated by activation of the plasma membrane involving increased intracellular calcium levels.^26^ Microvesicles, like exosomes, have unique lipid compositions, and are enriched in phosphatidylserine.^27^ Many protein markers are known to express on exosomes and microvesicles, including tetraspanins such as CD9, CD62 and CD81, membrane transporters and fusion proteins such as annexins, EV synthesis proteins such as TSG101, and other EV‐associated proteins.^25^, ^28^ Apoptotic bodies are predominantly secreted by cells experiencing apoptosis.^29^ They are, in general, observed to be larger than exosomes and microvesicles, and contain fragments of DNA and cell organelles from the apoptotic cells. These apoptotic bodies are formed during blebbing of the plasma membrane.^30^

### 1.4 EV signalling

EV signalling is an important mode of intercellular communication, given the myriad of bioactive molecules, including DNA, RNA, and proteins, which are carried and transferred by EVs. Tumour metastasis and progression are not an exception to this. EVs play a pivotal role in communicating between tumour cells and other cells in the tumour microenvironment; via EVs, highly metastatic tumour cells are able to transfer biomolecules to less malignant cells.^31^ Consequently, the less malignant cells receiving the EVs may start to display enhanced migratory and metastatic behaviour.^31^ An example is the avß3 integrin, which is upregulated during cancer progression and acknowledged to account for the migration of cancer cells, hence leading to metastasis of tumours. EVs are able to transfer avß3 integrins from tumorigenic cells to other cells, causing recipient cells to express malignant traits, increasing avß3‐dependent adhesion and migration.^32^

EV signalling is also capable of inducing the changes of tumour cell characteristics and behaviours, such as drug resistance. Challagundla and colleagues^33^ found that human monocyte‐derived EVs were able to transfer miR‐155 to neuroblastoma cells; upon EV‐mediated delivery, the miR‐155 directly targeted TERF1, which is a component of the shelterin complex and also a telomerase inhibitor. Increased cisplatin resistance was hence conferred to the neuroblastoma cells. Similarly, Chen et al showed that adriamycin and docetaxel resistance can be conferred by EV‐mediated microRNA transfer.^34^ Cell surface proteins of EVs contribute to drug resistance as well. Ko et al posited that small EVs (sEVs) promote angiogenesis via their EV‐surface‐specific isoform of vascular endothelial growth factor (VEGF), which cannot be neutralized by bevacizumab,^35^ the therapeutic monoclonal antibody against VEGF reported to be effective in many solid cancer treatments.^36^, ^37^, ^38^ The combination treatment of bevacizumab with a Hsp90 inhibitor, 17‐N‐allyamino‐17‐demothoxygeldanamycin (17AAG), can restore the bevacizumab sensitivity of EV surface VEGF and inhibit tumour growth in a breast cancer patient‐derived xenograft model through 17AAG localizing to microvesicles (MVs), binding VEGF_90K_ and releasing this VEGF, thus increasing the efficacy of bevacizumab.^39^

EVs may also play a role in immunosuppression allowing unchecked proliferation of tumours and subsequently, their metastasis. EV uptake by immune cell lineages dampens immune‐cell populations, reducing immune surveillance necessary for the body to recognize and kill tumour cells. A study by Wen et al^40^ demonstrated reductions in CD4 and CD8 T‐cell populations and attenuation of the cytotoxic capabilities of natural killer (NK) cells upon accumulation of breast tumour‐derived EVs in lung. This contributes to the pre‐metastatic niche, paving the way for subsequent metastases. PD‐L1 expression by tumour‐derived EVs can inhibit CD8 T‐cell proliferation, cytokine production and cytotoxicity,^41^ and in turn induce the proliferation of other immunosuppressive cell types, such as PD‐1 positive nonclassical monocytes (NCM).^42^

EVs are also responsible for reprogramming cellular metabolism, which is essential in providing energy for dissemination and proliferation of cancer cells, especially at distant sites where vasculature and nutrients are lacking.^43^ Cancer‐associated fibroblast (CAF)‐derived EVs inhibit mitochondrial oxidative phosphorylation, and shuttle de novo ‘off the shelf’ metabolites for cancer cells.^44^

These respective findings demonstrate that EV signalling plays an important role in cancer initiation and progression. Further discoveries pertaining to organ‐specific metastasis will be detailed in coming sections.

## 2 EXTRACELLULAR VESICLES IN ORGANOTROPIC METASTASIS

In the context of cancer metastasis and organotropism, EVs are best known to function by modulating the pre‐metastatic niche formation. Paget's ‘seed and soil’ hypothesis has established the conceptual architecture for organotropic metastasis studies.^45^ In brief, tumour cells are considered as ‘seeds’ while metastatic sites as ‘soil’. In order for the ‘seeds’ to grow in the new ‘soil’, the tumour cells and the microenvironment at the metastatic sites need to be compatible: the ‘seeds’ require intrinsic properties to expand and the ‘soil’ has to be congenial.^45^ Recent reports have shown that tumour cells at primary sites are able to signal resident cells at potential metastatic sites via EVs.^46^ Primary tumour cells secrete EVs into circulation as messengers even before their arrival at distant sites.^47^ Carrying bioactive molecules, EVs travel from primary tumour sites, arrive at the pre‐metastatic niches, and are internalized by resident cells there.^47^ After internalization, molecules transported by EVs participate in biological interactions in the recipient cells. For instance, the cargo delivered by cancer‐derived EVs encourage CAF formation, endothelial cell migration and angiogenesis.^48^ These are all important steps in forming a distant microenvironment allowing cancer development and progression. EVs derived from prostate cancer and mesothelioma cell lines were shown by Webber et al^49^ to express transforming growth factor‐beta (TGF‐β), triggering the TGF‐β/SMAD_3_ pathway in fibroblasts allowing their differentiation into myofibroblastic phenotypes.

Furthermore, it is not merely tumour‐derived EVs which are capable of inducing metastasis—EVs derived from other cell types, which interact with the primary tumour cells, can further enhance the metastatic potential of certain tumours. Janowska‐Wieczorek et al^50^ found that platelet‐derived EVs (pdEVs) chemoattract human lung cancer cells. pdEVs encourage metastasis through activation of MAPK p42/44 and AKT, and upregulate matrix metalloproteinases (MMPs), and also induce angiogenesis by upregulating VEGF, IL‐8 and HGF, in addition to MMPs. pdEVs are also capable of transferring platelet‐derived integrins, such as CD41, to lung cancer cells.

**Figure 1:**
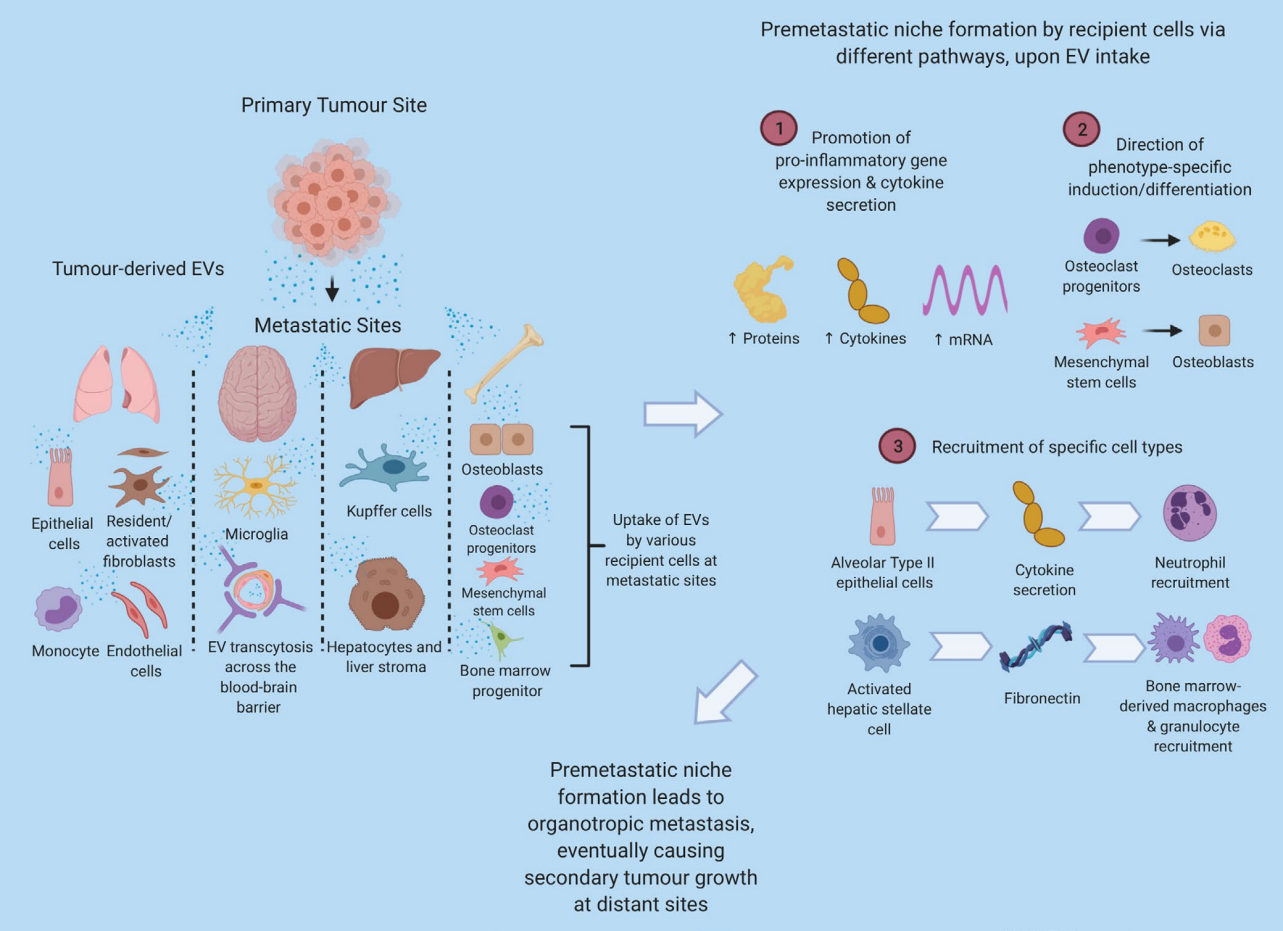
Extracellular vesicle (EV)‐mediated organotropic metastasis via pre‐metastatic niche modulation. Investigations showed that pre‐metastatic niche modulation via EVs plays a major role. Primary tumour cells disseminate EVs which can travel to distant metastatic sites and modulate resident or stroma cells in the pre‐metastatic microenvironment through various pathways, such as promotion of pro‐inflammatory gene expression and cytokine secretion, direction of phenotype‐specific induction/differentiation, and recruitment of specific cell types. The EV‐mediated modulation prepares a favourable pre‐metastatic niche at distance for metastatic tumour cells to reside in and grow

Thus, EVs are responsible for tuning the distant microenvironment into a piece of favourable ‘soil’ for the ‘seeds’ tumour cells to grow.^47^ A deeper understanding of EVs in site‐specific metastasis may unravel novel uses of EVs for diagnostic and therapeutic purposes. We next review recent studies investigating EV‐associated metastatic organotropism by organs where distant metastasis frequently happens—in the lungs, brain, bone and liver (Figure 1; Table 1).

**Table 1:** Summary of recent studies regarding extracellular vesicle (EV)‐mediated organotropic metastasis

Metastatic sites	EV donor cells	EV cargo	EV recipient cells	Downstream effects	References
Lung	Breast cancer and melanoma cells	Integrin α6 β1 and integrin α6 β4	Surfactant protein C‐positive lung epithelial cells	Upregulation of pro‐inflammatory gene expression S100A4, A6, A10, A11, A13, and A16	Hoshino et al^51^
	Breast cancer cells	miRNA‐125b	Pulmonary resident fibroblasts	Transformation of fibroblasts into activated phenotypes	Vu et al^52^
	Metastatic breast cancer cells	miRNA‐105	Endothelial cells	Targeting tight junction protein zonula occludens‐1 (ZO‐1), hampering endothelial monolayers’ barrier functions, and increasing vascular permeability	Zhou et al^55^
	Colorectal cancer cells	miRNA‐25‐3p	Endothelial cells	Targeting KLF2 and KLF4, and thus regulating VEGFR2, ZO‐1, occludin and CLDN5 expression to enhance vascular leakiness	Zeng et al^56^
	Breast cancer cells	Annexin A6	Lung tissues (unspecified)	Induction of CCL2 expression, and expansion of Ly6C^+^CCR2^+^ monocytes	Keklikoglou et al^57^
	Lung carcinoma cells	EV‐associated RNAs (unspecified)	Alveolar type II epithelial cells	Activation of TLR3, and increased cytokine secretion and neutrophil recruitment	Liu et al^58^
Brain	Breast cancer cells	Unspecified	Brain endothelial cells	Downregulation of rab7 expression to facilitate tumour‐derived EVs passing intact brain‐blood barrier through transcytosis	Morad et al^61^
	Breast cancer cells	miRNA‐181c	Brain endothelial cells	Downregulation of PDPK1, induction of cofilin‐modulated actin dynamics interruption and disruption of brain‐blood barrier integrity	Tominaga et al^62^
	Lung cancer and breast cancer cells	CEMIP	Brain endothelial and microglial cells	Upregulation of pro‐inflammatory cytokines coded by Ptgs2, Tnf and Ccl/Cxcl, leading to brain vascular niche remodelling	Rodrigues et al^63^
	Breast cancer cells	miRNA‐503	Microglial cells	Induction of M1‐M2 polarization and upregulation of immunosuppressive cytokines	Xing et al^64^
	Astrocytes	miRNA‐19a	Breast cancer tumour cells	Causing adaptive PTEN loss in cancer cells metastasized to the brain, and leading to tumour outgrowth through CCL2 upregulation and recruitment of IBA1‐expressing myeloid cells	Zhang et al^65^
Bone	Prostate cancer cells	EV‐associated RNA (both mRNAs and miRNAs)	Osteoblasts	Increased osteoblast viability and an enhanced growth environment	Probert et al^70^
	Lung adenocarcinoma cells	miRNA‐21	Osteoclast progenitors	Targeting of Pdcd4 resulting in increased osteoclastogenesis	Xu et al^72^
	Prostate cancer cells	miRNA‐940	Human mesenchymal stem cells (hMSCs)	Targeting of ARHGAP1 and FAM134A in hMSCs, resulting in osteoblastic phenotype differentiation	Hashimoto et al^74^
	Breast cancer cells	miRNA‐940	hMSCs	Osteogenic differentiation of hMSCs resulting in osteoblastic lesions in resultant metastatic tumour	Hashimoto et al^74^
	Melanoma cells	c‐MET	Bone marrow progenitor cells	Differentiation of bone marrow progenitor cells into pro‐vasculogenic phenotypes	Peinado et al^75^
Liver	Breast cancer and melanoma cells	Integrin α V β 5	F4/80‐positive Kupffer cells	Upregulation of pro‐inflammatory gene expression S100P, A8	Hoshino et al^51^
	Pancreatic ductal adenocarcinoma	MIF	Kupffer cells	Stimulation of TGFß production by Kupffer cells, and activation of hepatic stellate cells	Costa‐Silva et al^80^
	Colorectal cancer cells	miRNA‐21	Liver macrophages	Induction of IL‐6‐secreting phenotype via TLR7 pathways	Shao et al^81^
	Gastric cancer cells	EGFR	Liver stromal cells	Downregulation of miRNA‐26a/b leading to activation of hepatocyte growth factor	Zhang et al^82^
	Colorectal cancer cells	miRNA‐25‐3p	Endothelial cells	Targets KLF2 and KLF4, thus regulating VEGFR2, ZO‐1, occluding and CLDN5 expression	Zeng et al^56^

### 2.1 Lung‐tropic metastasis

Lung is a frequent metastatic site for breast, colorectal, and pancreas cancers as well as melanoma. Hoshino et al^51^ demonstrated that tumour‐derived, lung‐tropic EVs bear integrins α6β1 and α6β4 and are favourably taken up by lung fibroblasts and surfactant protein C‐expressing lung epithelial cells. Incorporation of such EVs by lung resident cells increase the expression of pro‐inflammatory genes *S100* and promote lung metastasis while blocking lung‐tropic EVs surface integrins using HYD‐1 peptides can inhibit the uptake by lung resident cells and reduce lung metastasis. This is consistent with our findings that tumour‐derived, microRNA‐125b‐containing EVs are preferentially taken up by pulmonary resident fibroblasts and transform them into activated phenotypes, and thus promote lung metastasis.^52^ Moreover, Ortiz et al^53^ reported the protective effect of cholesterol 25‐hydroxylase (CH25H) in healthy cells against pro‐metastatic education by tumour‐derived EVs. Interferon‐inducible CH25H hampers tumour‐derived EVs uptake by generating 25‐hydroxycholesterol. In vivo assays showed that inability to downregulate IFN receptor and *Ch25h* protects the lung from pre‐metastatic niche formation via ablating tumour‐derived EVs education, thus reducing melanoma lung metastasis. Surprisingly, reserpine, the classic anti‐hypertensive beta blocker, reduced lung metastasis by refusing tumour‐derived EVs education, which appears to be a potential novel therapeutic target against lung metastasis.

One of the most characteristic features of the lung is its abundance in capillaries, providing the physiological territories for tumours cells to arrest, adhere, extravasate, and grow.^54^ EVs are actively involved in influencing the lung vascular permeability as well. According to Zhou et al,^55^ microRNA‐105 can be transferred from metastatic breast cancer cells to endothelial cells via EV‐mediated cell communication, which hampers the barrier functions of endothelial monolayers, increases vascular permeability, and promotes metastasis in lung via targeting the tight junction protein zonula occludens‐1 (ZO‐1). Besides, microRNA‐105 in circulation has been proved to correlate with metastasis in patients with early‐stage breast cancer. Similarly, Zeng et al^56^ reported the vascular permeability‐enhancing and angiogenic function of microRNA‐25‐3p in colorectal cancer (CRC)‐derived EVs. The transfer of EV‐enclosed miR‐25‐3p to endothelial cells regulates the expression of VEGFR2, ZO‐1, occludin and CLDN5 via targeting KLF2 and KLF4, enhances vascular leakiness, and fosters CRC lung metastasis in mice. Higher levels of microRNA‐25‐3p in serum EVs from CRC patients are associated with increased risks of distant metastasis.

In addition to pulmonary vasculature and capillaries, immune cells such as monocytes and neutrophils are documented to dynamically participate in EV‐mediated pulmonary tropism as well. Keklikoglouv et al^57^ explored the undesired pro‐metastatic effects of cytotoxic chemotherapy in breast cancer. Cytotoxic chemotherapeutics can elicit tumour cells to secrete annexin A6‐enriched EVs and promote the expansion of Ly6C^+^CCR2^+^ monocytes on which the pro‐metastatic property of annexin A6‐enriched EVs relies. Alarmingly, breast cancer patients undergoing chemotherapy showed higher levels of annexin A6 in circulating EVs as compared to pre‐treatment conditions, implying the potential risks of chemotherapy‐related metastasis. Moreover, Liu et al^58^ demonstrated that tumour‐derived EVs can activate Toll‐like receptor (TLR) 3 and boost cytokine secretion in alveolar type II epithelial cells, prompting the recruitment of neutrophils and promoting lung metastasis. High levels of TLR3 expression and neutrophil infiltration in lung lesions are predictive of poor prognosis.

### 2.2 Brain‐tropic metastasis

Brain metastasis is mostly observed in breast, kidney, lung cancers, and melanoma. The distinctive biophysiological property that separates brain from other metastatic sites is the blood‐brain barrier (BBB), which is a highly selective border controlling substance permeability. The BBB consists of continuous capillary wells of endothelium cells joint by tight junction with astrocyte end feet ensheathing and pericytes embedded in the basement membrane.^59^ Intact BBB needs to be disrupted or compromised for tumour cells to metastasize to the brain.^60^ Recently, Morad and colleagues^61^ demonstrated that tumour‐derived EVs from breast cancer can cross intact BBB through transcytosis in vitro and in vivo. Specifically, breast cancer‐derived EVs enhance the relatively low rate of transcytosis under physiological conditions via downregulation of rab7 expression in brain endothelial cells. Tominaga et al^62^ also reported that the tumour‐derived, microRNA‐181c‐containing EVs can mediate pre‐metastatic modulation via disruption of BBB. Incorporation of microRNA‐181c‐containing EVs can cause the downregulation of PDPK1 and the subsequent cofilin‐modulated interruption of actin dynamics, resulting in breakdown of BBB integrity.

**Figure 2:**
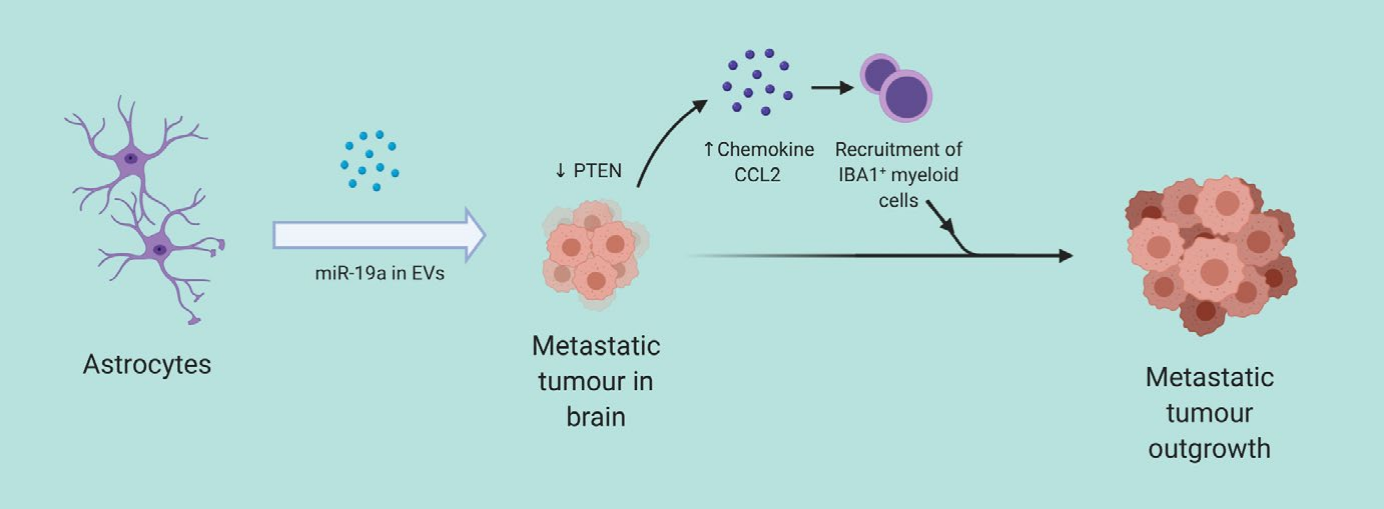
Stroma‐to‐tumour communication via EVs. Resident cells at metastatic sites can, in turn, secrete EVs for uptake by tumour cells and promote secondary tumour outgrowth. In this instance, astrocyte‐derived EVs containing microRNA‐19a can be internalized by metastatic tumour cells in brain, cause their downregulation of PTEN and promote the secretion of chemokine CCL2. CCL2 further recruits IBAB^+^ myeloid cells to facilitate the metastatic tumour outgrowth

Tumour cells and the brain pre‐metastatic microenvironment can have reciprocal crosstalk via EVs. On the one hand, tumour cells use EVs as messengers to modulate the brain pre‐metastatic niche. Rodrigues et al^63^ identified the distinct increase of cell migration‐inducing and hyaluronan‐binding protein (CEMIP) expression in brain‐tropic EVs in comparison to lung‐tropic or bone‐tropic EVs and demonstrated that ablation of CEMIP in tumour cells hinders brain metastasis. CEMIP‐expressing, brain‐tropic EVs prompts remodelling and inflammation in brain vasculature, upon the uptake by brain endothelial and microglial cells via increasing secretion of pro‐inflammatory cytokines. Elevated levels of CEMIP are also predictive of brain metastasis and poor prognosis in patients. In another study by Xing et al,^64^ microglial cells are also considered to be reprogrammed by tumour cells through EV crosstalk. The long non‐coding RNA XIST was found to be of lower levels in breast cancer patients with brain metastasis and downregulation of XIST enhances tumour cell malignancy and induces brain metastasis in vivo. Ablation of XIST in tumour cells also triggers microRNA‐503 secretion in EVs, whose internalization by microglia cells induces M1‐M2 polarization and turns up levels of immunosuppressive cytokines. On the other hand, cells from the brain pre‐metastatic niche can in return signal tumour cells via EVs (Figure 2). Zhang et al^65^ observed the loss of expression of tumour suppressor gene PTEN in tumour cells only when they metastasized to the brain and were in the brain microenvironment. Additional experiments revealed that astrocytes, known to secrete cytokines and growth factors, can disseminate EVs that contain PTEN‐targeting microRNA‐19a, which causes the adaptive PTEN loss and promotes the outgrowth in brain‐metastatic tumour cells by recruiting IBA1‐expressing myeloid cells. The bilateral communication between tumour cells and the brain pre‐metastatic niche acts synergistically to promote brain metastasis and tumour outgrowth. Breaking the crosstalk cycle may emerge as an attractive novel therapeutic strategy.

### 2.3 Bone‐tropic metastasis

Tumour cells from melanoma, prostate, lung, and breast cancers can metastasize to bone. One unique feature of the bone microenvironment is the distinct composition and dynamics of bone‐resorbing osteoclasts, bone‐forming osteoblasts, and the bone‐residing osteocytes.^66^ Different types of cancers present different bone metastasis patterns. For instance, prostate cancer patients usually manifest an osteoblast phenotype in bone metastatic lesion^67^ while osteoclastic lesion is prominent in breast or lung cancer patients with bone metastasis.^68^, ^69^ Probert and colleagues^70^ showed that tumour‐derived EVs from metastatic prostate cancer cells are enriched in RNAs involved in pathways such as cell surface signalling and protein translation and are delivered to osteoblasts, leading to an influence on endogenous transcript abundance in osteoblasts. Co‐culture with metastatic prostate cancer EVs results in osteoblasts showing enhanced viability and generating a preferential environment that supports tumour cell growth. Known to be crucial in metastasis‐associated osteolysis,^71^ osteoclastogenesis is found to be mediated by tumour‐derived EVs from lung adenocarcinoma cells by Xu et al.^72^ EVs from lung adenocarcinoma cells contain high levels of microRNA‐21 which can be transferred to osteoclast progenitor via EVs, and promote osteoclastogenesis by targeting Pdcd4. In brief, osteoblast‐osteoclast interactions are complex and dynamic^73^ but the involvement of tumour‐derived EVs in the interactions is not well understood and warrants further investigations.

Tumour‐derived EVs also alter the pre‐metastatic bone microenvironment though inducing phenotype‐specific differentiation. A recent study by Hashimoto and colleagues^74^ found that prostate cancer cells‐derived EVs contain microRNA‐940 which can target ARHGAP1 and FAM134A in human mesenchymal stem cells and induce osteoblastic phenotype differentiation. Surprisingly, even in breast cancer cells that are considered to generate osteoclastic lesion, overexpression of microRNA‐940 can induce osteoblastic lesion in the bone microenvironment in an EV‐dependant manner. Similarly, melanoma cell‐derived EVs target bone marrow progenitor cells and convert them towards a pro‐vasculogenic phenotype favourable for metastasis through c‐MET, as reported by Peinado et al.^75^

### 2.4 Liver‐tropic metastasis

Liver metastasis is primarily described in lung, breast, and gastrointestinal cancers. One of the most studied liver resident cell types that interact with tumour‐derived EVs are Kupffer cells. Kupffer cells are specialized macrophages residing in liver sinusoids next to endothelial cells.^76^ Anatomically, Kupffer cells stand a good chance of contact with circulating tumour‐derived EVs. Both the hepatic artery and the hepatic portal vein provide dual and ample blood supply to the liver.^77^ In addition, there are fenestrae or open pores in the liver sinusoidal endothelial lining, making it easier for materials in circulation such as EVs to pass through.^78^ Functionally, Kupffer cells act as an intermediator in the signalling: on the one hand, they perform active functions in engulfing particles; on the other, they secrete many bioactive species such as cytokines to affect their neighbouring cells.^79^ Detailed by Costa‐Silva et al,^80^ tumour‐derived EVs from pancreatic ductal adenocarcinoma express high level of migration inhibitory factor (MIF) and are preferentially taken up by Kupffer cells, which results in increased TGFβ production by Kupffer cells and activates hepatic stellate cells. Activated hepatic stellate cells produce high level of fibronectin and in return vigorously recruit bone marrow‐derived macrophages and granulocytes to the liver for pre‐metastatic niche formation. Downregulation of MIF in tumour‐derived EVs abolishes initiation of liver pre‐metastatic modulation and levels of MIF in EVs from pancreatic ductal adenocarcinoma patients are useful for early metastasis detection. Liver tropism is associated with integrin αVβ5 on tumour‐derived EVs as reported by Hoshino et al^51^ These integrin αVβ5‐positive EVs are internalized by Kupffer cells, cause an upregulation of members from pro‐inflammatory genes *S100* and contribute to pre‐metastatic niche formation. Furthermore, blocking integrin αVβ5 on EVs using either RGD peptide or anti‐integrin αvβ5 antibody significantly diminishes EVs uptake by Kupffer cells and formation of liver pre‐metastatic niche. Shao and colleagues^81^ demonstrated the importance of microRNA‐21‐TLR7 axis in the EV‐mediated crosstalk in the liver‐specific metastasis of CRC. CRC‐derived EVs are enriched in microRNA‐21 and uptake of such EVs directs liver macrophages towards an interleukin‐6‐secreting phenotype through TLR7 pathways while silencing microRNA‐21 in CRC‐derived EVs or downregulation of TLR7 in liver macrophages ablates the pro‐inflammatory induction of liver macrophages. Levels of microRNA‐21 in circulating EVs from CRC patients are shown to correlate with liver metastasis.

On top of Kupffer cells, liver stromal cells are also finetuned by tumour‐derived EVs. Zhang et al^82^ identified the translocation of epidermal growth factor receptor (EGFR) in hepatotropic metastasis of gastric cancer. Gastric cancer cells deliver surface EGFR which is later integrated to plasma membrane of liver stromal cells through tumour‐derived EVs, resulting in activation of hepatocyte growth factor, a c‐MET ligand, via downregulation of miR‐26a/b, and modulation of a fertile microenvironment susceptible to liver metastasis. As discussed earlier in the lung tropism section, the pro‐vascular leakiness and pro‐angiogenic property of microRNA‐25‐3p in CRC‐originating EVs also contribute to liver metastasis in vivo.^56^

## 3 EVS AS DIAGNOSTIC AND PROGNOSTIC BIOMARKERS IN CANCER METASTASIS

The transport and delivery of the bioactive cargo is fundamental to EV‐mediated intercellular communication and has motivated a number of translational studies examining and exploiting EVs for metastasis‐associated diagnostic, prognostic, and potentially therapeutic applications.

From the perspective of diagnostic and prognostic studies, the secretion of EVs by donor cells and uptake by recipient cells have been widely observed in a broad spectrum of human diseases, including cancers,^20^ cardiovascular conditions,^83^ metabolic dysfunctions,^84^ autoimmune disorders,^85^ and neurological diseases.^86^ Profiling EVs using sequencing and mass spectrometry empowers us to decode the molecular messages that the diseased cells try to send. Since EVs carry molecular features from parental cells that are highly biorelevant, their molecular profiles may be of unparalleled diagnostic and prognostic values as compared to other clinical benchmarks.^87^ The abundance of EVs in biofluids provides the possibility to readily evaluate EVs in a minimally invasive manner.^88^ EVs can serve as dynamic snapshots of the diseased parental cells to reflect real‐time functional changes, which likely occur far before possible detection by observation of morphological changes or by imaging. In terms of biomedical and translational research, a better understanding of EVs and their cargo can potentially reveal the molecular mechanisms involved in these diseases (Table 2).

**Table 2:** Diagnostic and prognostic applications of EVs in cancer metastasis

Categories of detection	Sources of the detected EVs	EV cargo	Detection methods	Implications of study	References
Detection of EV proteins	Circulating EVs from CRC patients	CD147	ExoScreen: amplified luminescent proximity homogenous assay with photosensitiser beads	ExoScreen is potentially superior to immunoblotting and ELISA in EV detection. CD147 detection can detect early CRC better than current biomarkers, CA19‐9 and CEA.	Yoshioka et al^89^
	Prostate, lung, liver and colonic cancer cells	Phosphoproteins within EVs	Identification of SERS peak due to oscillations of the P‐O bond during protein phosphorylation	EV monitoring can be performed on patients’ serum.	Dong et al^90^
	Plasma of CRC patients	ITGBL1	ELISA	ITGBL1‐rich EVs in plasma correlate with CRC metastasis	Ji et al^91^
	Ascitic fluid of ovarian cancer patients	TGM2, U2AF1, U2AF2, HNRHPU	Proteome analysis with and without CPLL treatment, and analysis of silylated samples via mass spectrometry	Diagnostic of malignant ascites in ovarian cancer	Shender et al^92^
	Serum of stage IV melanoma patients	MIA, S100B	Immunoblotting and ELISA for detecting MIA and S100B	Raised MIA and S100B have diagnostic values and are associated with shorter median survival	Alegre et al^93^
	NSCLC patients post‐chemoradiation	Tspan8	SEM and NanoSight particle‐tracking paired with Western blot analysis to detect EV markers	Tspan8 level is predictive of subsequent distant metastasis	Liu et al^94^
	Serum of pancreatic cancer patients	GPC1‐positve crExo	Mass spectrometry	GPC1‐positve crExo detection surpasses MRI in early pancreatic cancer, and is predictive of survival	Melo et al^95^
Detection of EV nucleic acids	CSF of medulloblastoma (MB) patients	miR‐1290, miR‐125a/b	Microarray analysis with RT‐PCR analysis	miRNAs present in CSF of MB patients are potential biomarkers of disease	Shalaby et al^99^
	Serum of HCC patients	miR‐638	qRT‐PCR analysis	Lower serum EV miR‐638 predicts poorer prognosis in HCC patients	Shi et al^100^
	Plasma from metastatic prostate cancer patients	miR‐375, miR‐141	qRT‐PCR verification and analysis	miR‐375 and miR‐141 can differentiate aggressive tumours from more indolent ones and those less likely to metastasize	Bryant et al^101^
	Peripheral samples of glioblastoma patients	HOTAIR	qRT‐PCR with Western blot analysis for verification of CD63 on EVs	HOTAIR levels can predict treatment response of tumours to BET inhibitors	Tan et al^102^
	Plasma from cancer patients with bone metastasis	miRNAs associated with bone metastasis	Next‐generation sequencing as well as survival and progression analysis	Identification of tdEV‐associated miRNAs which contributes to bone metastasis and progression	Giavaresi et al (NCT03895216)
Detection of total EV counts	Castration‐resistant prostate cancer cells; metastatic colorectal cancer; metastatic breast cancer	NA (EV counts were used rather than EV cargo detection)	ACCEPT software for quantifying tdEVs from digitally stored CellSearch® images	Tumour‐derived EV counts have equivalent prognostic value as CTCs, and can further stratify prognosis for patients with favourable CTCs	Nanou et al^15^

The overall EV count can provide prognostic value for cancer patients. A recent study by Nanou et al^15^ showed that tumour‐derived EV (tdEV) counts provide equivalent prognostic value as circulating tumour cell (CTC) count in castration‐resistant prostate cancer, metastatic colorectal cancer and metastatic breast cancer. Furthermore, in patients with these cancers, the tdEV count further stratifies patients with favourable CTC counts.

Detection of tumour‐derived EVs can also be done through identification of their cargo. Using EV‐associated proteins as biomarkers is one method of detecting EVs in patients. Yoshioka et al developed ExoScreen^89^ to detect EVs in serum, based on an amplified luminescent proximity homogenous assay using photosensitiser beads, without the need for purification steps. CD147 is raised in serum EVs from CRC patients—this rise is detectable earlier than current biomarkers used for CRC diagnosis such as carcinoembryonic antigen (CEA) and carbohydrate antigen 19‐9 (CA19‐9). The authors also showed that the ExoScreen platform uses as little as one microlitre of EV‐containing culture medium for detection and has superior results to conventional immunoblotting and ELISA.

Another method was devised by Dong and colleagues^90^ to detect EVs by using the surface‐enhanced Raman scattering (SERS) technique to monitor the protein phosphorylation process in EVs. A 3D gold‐coated TiO2 macroporous inversion opal (MIO) structure was developed that can coordinate the interaction of the laser used, sample, and the SERS substrate. 1087cm^‐1^ SERS peaks of the EVs from prostate, lung, liver and colonic cancer cell lines were double that in normal cells. These changes were apparent from EVs taken from prostate cancer patients’ plasma as well, showing promise in cancer diagnostics via EV monitoring.

Specific EV proteins associated with certain cancers have been identified as possible biomarkers for cancer diagnosis. Ji et al showed that integrin beta‐like 1 (ITGBL1) was highly expressed in primary CRC and metastatic sites in comparison to normal tissues, and its high levels were also detectable within plasma EVs of CRC patients.^91^ Similarly, levels of TGM2, an extracellular matrix protein, U2AF1, U2AF2, and HNRHPU, which are intracellular spliceosomal proteins, were raised in EVs from ascitic fluid of ovarian cancer patients compared to normal ascitic fluid.^92^ In some cases, the level of these biomarkers can be used for prognostic purposes—in stage IV melanoma patients, the melanoma biomarkers MIA and S100B are raised and detectable in EVs from their sera; raised levels of these biomarkers are also associated with shorter median survival.^93^ In a different study on non‐small cell lung cancer (NSCLC), Liu et al^94^ recognized that in NSCLC patients who had gone through curative chemoradiation, levels of the protein tetraspasnin‐8 (Tspan8) in EVs were predictive of the likelihood of subsequent distant metastasis. These various studies show the potential application of EV detection as alternatives to current cancer diagnostic and prognostic tools. In fact, in some cases such as pancreatic cancer, EV protein detection may even outdo the capabilities of current technologies. Glypican‐1 (GPC1), a cell surface proteoglycan, was found to enrich circulating EVs (crEVs) in pancreatic cancer patients, and GPC1‐positve crEVs levels are predictive of disease‐specific survival.^95^ On top of that, GPC1‐positvecr EVs levels lead tumour growth, and are detectable before pancreatic masses can be detected by MRI.

Other techniques rely on identification and detection of the nucleic acids contained within tumour‐derived EVs. Since Kahlert et al showed that tumour‐derived EVs carry KRAS and P53 mutations from to their parental cancer cell genomic mutations,^96^ identifying known DNA mutations within serum EVs may be helpful to cancer diagnosis. In addition, with the availability of high sensitivity PCR‐detection methods, detecting the nucleic acid components of EVs is highly plausible and extremely valuable. Möhrmann et al^97^ made use of next‐generation sequencing and droplet digital PCR techniques to analyse EV‐associated DNA (evDNA) obtained from the plasma of cancer patients with BRAF, KRAS or EGFR mutations. Patients with lower evDNA mutation allelic frequency (MAF) were associated with longer median survival and time to treatment failure. EV microRNA is another promising biomarker, having been identified in a range of body fluids.^98^ More importantly, EV microRNA biomarkers respond to the disease states, and even return to normal levels after surgical resection of the tumour. This reflects the possibility of using EVs as biomarkers to track disease progression. Hence, monitoring these biomarkers in patients may allow us to differentiate tumours which are metastatic, recurrent, or high‐grade from those which are not. Shalaby et al^99^ conducted a study on medulloblastoma (MB) patients and found 3 metastasis‐associated extracellular microRNAs (miR‐1290, miR‐125a, miR‐125b) over‐represented in culture mediums from metastasis‐related MB cell lines that were also significantly enriched in their cerebrospinal fluid (CSF). In HCC patients, EV‐associated miR‐638 inhibits HCC cell proliferation and influences the overall progression of the tumour, with lower levels of serum EV‐associated miR‐638 predicting poorer prognosis in these HCC patients.^100^

In some cases, the associations we can make between certain genetic fragments and cancers may better characterize patients’ condition and hence affect their treatment plan. Bryant et al^101^ verified EV miR‐375 and miR‐141 as biomarkers of recurrent metastatic prostate cancer—picking out these miRNAs in patients’ EV samples can help clinicians differentiate aggressive prostatic tumours with high likelihood of metastasis from more indolent tumours or those less likely to metastasize. Tan et al demonstrated that EV cargo can also predict treatment outcomes. Serum HOX Transcript Antisense Intergenic RNA (HOTAIR) in EVs is not only a good diagnostic biomarker in glioblastoma patients, as it is detectable in glioblastoma (GBM) patients’ peripheral samples but absent in healthy patients, but also prognostic in its evaluation of treatment response of BET inhibitors on the tumour.^102^ Currently, there is an ongoing clinical trial (NCT03895216) aiming to identify EV‐microRNAs associated with bone metastasis in cancer patients. The above studies demonstrate the diagnostic as well as prognostic values of genetic information carried by EVs in determining disease status and treatment outcomes in cancer patients.

## 4 NOVEL EV‐ASSOCIATED THERAPEUTIC STRATEGIES AGAINST CANCER METASTASIS

From a therapeutic point of view, EVs are emerging as a novel drug delivery system thanks to their inherent biocompatibility and biosafety, in contrast to other synthetic nanoparticles.^103^ Therapeutic aspects of EVs can be divided into two classes, those that use unmodified EVs and those that rely upon engineered EVs. Instances of unmodified EVs as therapeutics include mesenchymal stem cell‐derived EVs^104^ and induced pluripotent stem cell‐derived EVs^105^ in tissue repair. However, since unmodified EVs have usually shown limited efficacies, researchers are now engineering naïve EVs by introducing therapeutic molecules into EVs and/or modifying EVs surface components in an attempt to improve their efficacies, by increasing EVs circulation half‐life, tissue targeting specificity, or designated drug concentration.^106^ One instance of this was the encapsulation of oncolytic adenoviruses (OAs) within bioengineered cell membrane nanovesicles (BCMNs).^107^ These BCMNs protected the OAs surface from anti‐AD5 immunity, allowing the virus to evade the innate immune system, infect tumour cells and ultimately lyse them. Modification of the EVs may also increase their uptake at target sites. Nakase et al^108^ modified EVs with octaarginine peptide, an arginine‐rich cell‐penetrating peptide, resulting in increased micropinocytosis by cancer‐related receptors. Increased accumulation within the target site garners a greater therapeutic effect. However, altering EVs may cause them to take on different shapes and sizes, and the alteration process may damage the membrane, affecting subsequent efficacy.^109^

While EVs can be utilized as a novel drug delivery system, there is also the recognition that native EVs, especially those which are tumour‐derived, can hasten cancer progression and metastasis. Hence, there have been several strategies developed to counter the effects of these EVs: inhibiting secretion of tumour‐derived EVs; removing circulating tumour‐derived EVs from circulation; preventing uptake of tumour‐derived EVs at specific sites or organs; and utilizing EVs from other sources as adjunctive cancer therapy (Table 3).

**Table 3:** Novel EV‐associated therapeutic strategies against cancer metastasis

Therapeutic means	Interventions performed	Implications of study	References
Inhibiting tdEV secretion	Administration of sulfisoxazole	Diminishing sEV biogenesis and secretion through interference with ETA and ESCRT‐dependent downregulation of CD63, RAB27a and RAB7	Im et al^110^
	Utilization of WRG28 therapy	WRG28 inhibits DDR2, which is important in breast cancer metastasis and found abundant in tdEVs, to halt tumour‐microenvironment interaction	Grither et al^111^
Blocking the uptake of tdEVs	Administration of anti‐CD9, anti‐CD63 antibodies	Antibodies targeting specific EV proteins can inhibit EV uptake	Nishida‐Aoki et al^114^
	Administration of tocilizumab	Tocilizumab neutralizes IL‐6 produced by tumour EV‐educated mesenchymal stem cells in osteosarcoma	Baglio et al^115^
	RGD and HYD‐1 peptides	RGD and HYD‐1 block integrins on tumour‐derived EVs responsible for lung‐ and liver‐tropic metastasis (α6β1, α6β4, and αVβ5)	Hoshino et al^51^
	TC I‐15	TC I‐15 blocks integrin α2β1‐mediated EV uptake by lung fibroblasts	Kong et al^116^
	R243	R243, a CCR8 inhibitor, halts EV uptake by GBM cells, and thus inhibits the induction of temozolomide‐resistant phenotypes	Berenguer et al^117^
Removal of tdEVs from circulation	EGFR‐targeting aptamers functionalized with mesoporous silica nanoparticles (MSN‐AP)	Binding between MSN‐AP and lung cancer‐derived EVs expressing EGFR allows hepatic uptake and subsequent elimination of cancerous EVs from circulation	Xie et al^118^
EVs to improve the efficacy of immunotherapy and cancer vaccine	DC‐derived EVs (Dex)	Stimulation of T‐cell responses and conversion of tumours into stronger immunogenic targets.	Pitt et al^119^
	Incorporation of fusion‐competent G protein of VSV into ‘exosome‐like vesicles’ (ELVs)	Increased uptake of ELVs by DCs, enhanced DC co‐stimulatory and effector functions, and acceleration of antigen internalization and presentation	Temchura et al^120^
	Macrophages (M1)‐derived exosomes	Boosted efficacy of Trp2 vaccine through increased accumulation of immune cells in tumours, and inhibition of tumour growth	Cheng et al^121^

As discussed throughout this paper, tumour‐derived EVs are responsible for cancer progression and metastasis by allowing crosstalk between tumour cells and other cell types. Inhibiting tumours from secreting these malicious EVs may hinder disease progression. Im et al identified sulfisoxazole (SFX) as capable of diminishing small EV (sEV) biogenesis and secretion through the ESCRT‐dependent mechanism.^110^ SFX downregulates many components of the ESCRT machinery and suppresses microphthalmia‐associated transcription factor (MITF), and the downstream targets, such as reduced CD63, RAB27a and RAB7, which lead to reduced sEV biogenesis. In addition, SFX interferes with endothelin receptor A (ETA), reducing both the number of sEVs secreted as well as the protein amounts from these sEVs. In a different study, Grither et al^111^ utilized WRG28, a small molecule inhibitor of discoidin domain receptor 2 (DDR2) extracellular domain, to suppress tumour‐microenvironment interaction, tumour invasion, and further lung metastatic colonization by breast cancer cells. DDR2 is a collagen‐binding receptor tyrosine kinase (RTK) critical for breast cancer metastasis through multiple mechanisms including mediating integrin‐mediated mechanotransduction in CAFs,^112^ and have been found to be abundant in many cancer‐derived EVs.^113^

An alternative therapeutic approach aimed at EV function inhibition is by blocking the uptake of tumour‐derived EVs at their target sites and thus halting their downstream actions. Nishida‐Aoki et al utilized anti‐CD9 and anti‐CD63 antibodies to inhibit general EV uptake across multiple organs in mice.^114^ The administration of these antibodies reduced overall rates of metastasis. Although these same antibodies cannot be applied to humans as CD9 and CD63 are ubiquitous in the human body, the study demonstrates the pivotal proof of concept that neutralizing antibodies are capable of halting EV uptake, thus reducing rates of metastasis. Baglio et al achieved such an effect by administering tocilizumab, an therapeutic monoclonal antibody against IL‐6R, in treatment of osteosarcoma.^115^ The team showed IL‐6 production by tumour EV‐educated mesenchymal stem cells (TEMSC) to be responsible for stimulating tumour growth. The tumour‐promoting effects of TEMSCs could in turn be abrogated by tocilizumab. Blocking EV uptake can also be achieved at sites of likely metastasis. As earlier discussed, Hoshino et al^51^ demonstrated the capability of RGD and HYD‐1 peptides in blocking integrins to inhibit lung‐ and liver‐tropic metastatic EV uptake, thereby reducing metastasis to these organs. Similar methods of blocking integrins were used by Kong et al^116^ in reducing salivary adenoid cystic carcinoma metastasis to the lung. TC I‐15, a potent α2β1 integrin inhibitor, was shown capable of blocking integrin B1‐mediated EV uptake by lung fibroblasts, hence attenuating pre‐metastatic niche formation in the lung and subsequently, metastasis. In some cases, blocking EV uptake may prevent the tumour from expressing drug‐resistant phenotypes and thus improve treatment outcomes. Tumour‐derived EV uptake can induce temozolomide (TMZ) resistance in GBM patients.^117^ R243, a small molecule CCR8 inhibitor, can halt EV uptake by GBM cells and thus inhibiting the EV‐induced phenotypes; pre‐treatment with R243 improved tumour response to TMZ. These studies show that blocking uptake of tdEVs proves to be an effective way to inhibit tumour growth as well as metastasis to other organs.

Tumour‐derived EVs have been discussed with regards to the formation of the pre‐metastatic niche; removing these EVs from the patient's circulation is a relatively intuitive method to prevent the onset of metastasis. Xie et al^118^ targeted lung cancer‐derived EVs expressing EGFR (A‐Exo) with EGFR‐targeting aptamers functionalized with mesoporous silica nanoparticles (MSNs). Intermolecular binding forces between A‐Exo and MSN‐AP allow the conjugate (MSN‐Exo) to be rapidly taken up by liver hepatocytes and quickly excreted into the gastrointestinal tract via the bile duct, effectively eliminating A‐Exo from circulation.

EVs from other sources may also be used to improve the efficacy of cancer vaccines. For instance, dendritic cell (DC)‐derived EVs (DC‐EVs) have been used as maintenance immunotherapy. Via direct and indirect routes of stimulation, DC‐EVs and other antigen presenting cell (APC)‐derived EVs stimulate T‐cell responses.^119^ Furthermore, DC‐EVs uptake by tumour cells may potentially convert these tumours into stronger immunogenic targets, improving therapeutic response to other therapeutic agents. Temchura et al^120^ incorporated fusion‐competent G protein of vesicular stomatitis virus into ‘exosome‐like vesicles’ (ELVs), achieving increased uptake of these ELVs by DCs. Furthermore, these unique VSV‐G containing EVs are able to enhance the co‐stimulatory functions of DCs, induce their effector functions and accelerate the process of antigen internalization and presentation. By doing so, the immunostimulatory effects of EV vaccines are improved. Cheng et al^121^ also demonstrated the use of EVs as adjuvant therapy to boost the efficacy of the lipid calcium phosphate (LCP) nanoparticle‐encapsulated Trp2 vaccine. EVs derived from macrophages of the M1 phenotype stimulated pro‐inflammatory cytokine production, in turn allowing the cancer vaccine to induce a stronger cytotoxic T‐cell response. Accumulation of immune cells in tumours increased, and tumour growth was significantly inhibited.

In brief, researchers are utilizing EVs as promising therapeutic agents against cancer—either taking advantage of their unique properties, or actively countering their role in tumour progression and metastasis. The above section represents a recent and novel classification of the various studies done in this field. Further research will uncover more potential therapeutic applications of EVs in cancer therapy.

## 5 SUMMARIES AND FUTURE PERSPECTIVES

To summarize, organotropic metastasis involves the convergence of many complex biological interactions and processes, orchestrated by a combination of anatomical, pathophysiological, genetic and biochemical factors. In this context, tumour‐derived EVs function, largely, through pre‐metastatic niche establishment via intercellular communication with stromal cells within the niche. Primary tumour cells disseminate EVs which can travel to distant metastatic locations even prior to the final arrival of tumour cells. Bioactive cargo transported by EVs plays an essential part in metastasis and organotropism with EVs surface proteins frequently identified to be essential for recognition and internalization by recipient cells and EV‐enclosed microRNAs vital for signalling initiation in recipient cells. Upon uptake at distant sites, EVs modulate cells in the pre‐metastatic microenvironment through many pathways such as promotion of pro‐inflammatory genes expression and cytokines secretion, direction of phenotype‐specific induction or differentiation, and recruitment of specific cell types. The pre‐metastatic education by EVs turns the hostile or neutral microenvironment at distance into a piece of favourable and fertile ‘soil’ for the ‘seeding’ metastatic tumour cells to reside in and grow. Of note, the reciprocal crosstalk via EVs has been gradually recognized.^65^ Resident cells at metastatic sites can secrete EVs for uptake by tumour cells and promote secondary tumour outgrowth.^65^

**Figure 3:**
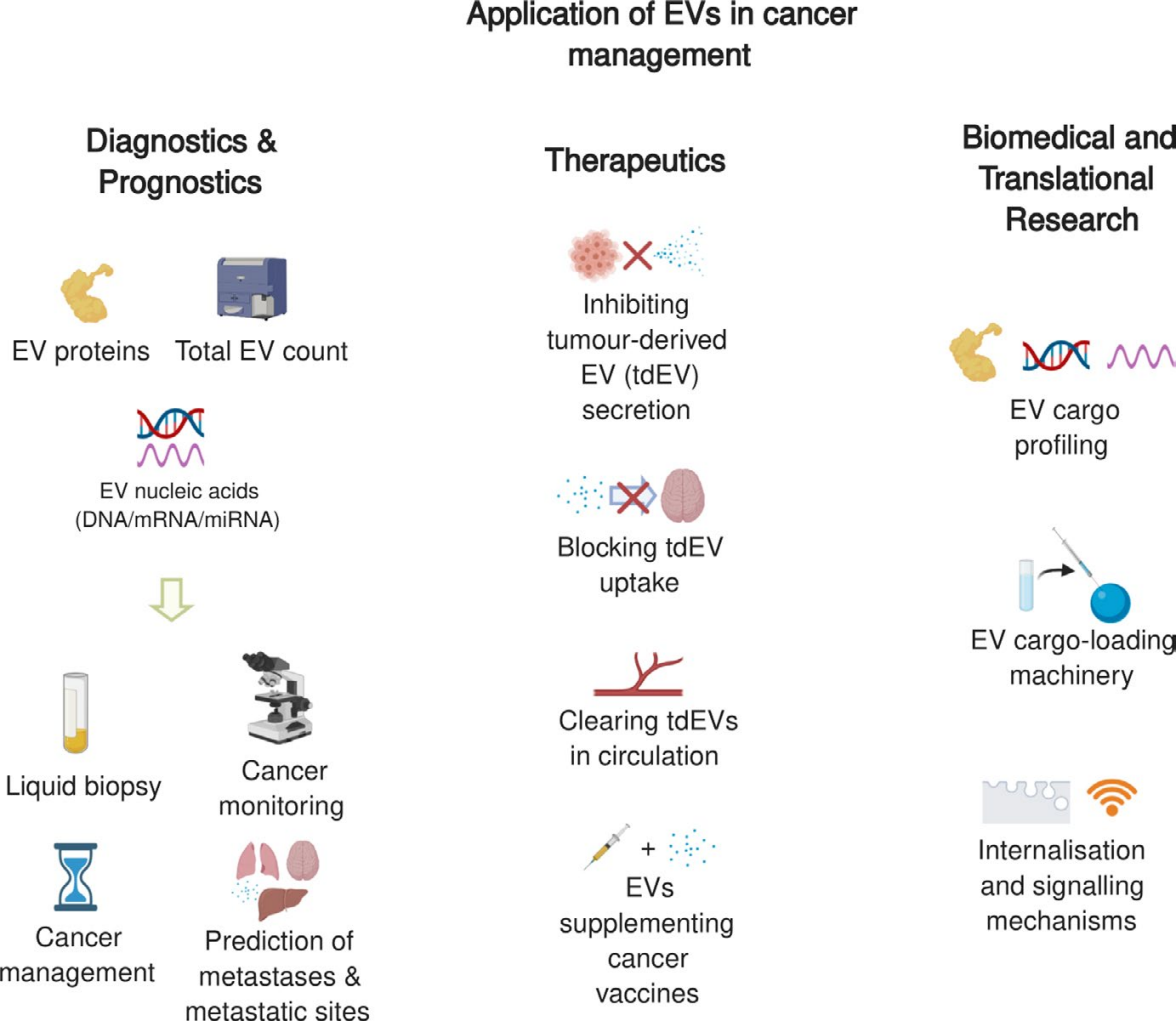
Applications of organotropic metastasis‐associated EVs. These metastatic and organotropic aspects of tumour‐derived EVs can be of great diagnostic, prognostic, and therapeutic potentials and of emerging interests in biomedical and translational research

With many of the current studies, we are gaining an increasingly improved understanding of how EVs promote metastasis and organotropism. The translation of this knowledge can be highly beneficial to cancer management in the clinic (Figure 3). From a diagnostic and prognostic point of view, the profiling of bioactive cargo carried by EVs can be informative of cancer status as circulating EVs are dynamic snapshots of the originating tumour cells, which can be collected in a minimally invasive manner.^87^ When a risk of either site‐specific or general metastasis is suspected through detection of EVs, more frequent monitoring and follow‐up or more radical treatment regimens can be considered. From a therapeutic perspective, counteracting the action of tumour‐derived EVs may be useful in minimizing metastasis. Further biomedical investigations of novel and less known topics are still warranted, such as the loading machinery of the bioactive cargo into EVs, inhibition of EVs function in action, and internalization mechanism and signalling initiation upon uptake. We believe that emerging novel techniques in EV research, such as label‐free EV visualization^122^ and blood oncogenic EV elimination,^118^ will surely deepen our understanding and accelerate the clinical translation of EVs in organotropic metastasis.

## CONFLICT OF INTEREST

MTNL is a cofounder, advisor and shareholder of Carmine Therapeutics, a red blood cell extracellular vesicle company. MTNL and AG are among the co‐inventors of the US provisional patent US 63/000,468 "Methods of delivering agents to immune cells". Other authors declare no conflict of interest.

## AUTHORS' CONTRIBUTIONS

MZ, JYAC, LX and DXZ searched the literature and drafted the manuscript. JYAC and LX visualized the artworks with the conceptual framework from MZ and DXZ. DXZ, AG and MTNL provided guidance and advice and edited the manuscript. All authors read and approved the final manuscript.

## Data Availability

Original data can be found in the corresponding references. No new datasets were generated for this review.
